# Changes in Muscle Pattern Activity during the Asymmetric Flat Bench Press (Offset Training)

**DOI:** 10.3390/ijerph17113912

**Published:** 2020-06-01

**Authors:** Jakub Jarosz, Artur Gołaś, Michal Krzysztofik, Patryk Matykiewicz, Katarzyna Strońska, Adam Zając, Adam Maszczyk

**Affiliations:** Institute of Sport Sciences, The Jerzy Kukuczka Academy of Physical Education in Katowice, ul. Mikolowska 72a, 40-065 Katowice, Poland; jaroszjakub88@gmail.com (J.J.); a.golas@awf.katowice.pl (A.G.); pmatyk@gmail.com (P.M.); k.stronska@awf.katowice.pl (K.S.); a.zajac@awf.katowice.pl (A.Z.); a.maszczyk@awf.katowice.pl (A.M.)

**Keywords:** bilateral movement, injury and prevention, performance, resistance exercise

## Abstract

Background: This study aimed to compare the muscle activity between the symmetric and selected asymmetric loads (2.5%; 5% and 7.5% differences in load position between sides of the bar) during the flat bench press (BP) exercise at 70%1RM. The study included 10 resistance-trained males (25.3 ± 2.3 years; 82.9 ± 6.9 kg; 177.8 ± 4.5 cm; 1RM BP: 104.5 ± 8.6 kg; experience: 5.6 ± 1.5 years). Methods: To assess the differences in muscle activity between both sides of the body and load placement, the participants performed several attempts of the BP with symmetric and asymmetric load at 70%1RM in a random order (symmetric; 2.5%; 5% and 7.5% differences in load position between sides of the bar). Peak muscle activity of dominant and non-dominant body-side was recorded for the pectoralis major (PM), anterior deltoid (AD), and the long head of the triceps brachii (TB). Results: A two-way repeated-measures analysis of variance (ANOVA) indicated a statistically significant main interaction between side and load (*p* < 0.01) for AD, PM and TB muscles. Conclusion: The results of this study showed that asymmetrically loaded BP leads to significantly higher muscle activity on the loaded side of the body. The offset training method during bilateral resistance exercise may be an effective and simple approach for reductions in muscle imbalances and improvement in bilateral exercise performance.

## 1. Introduction

In competitive sports, inter-limb asymmetry is often observed due to repetitive overuse of the active and passive movement apparatus [1]. Demands of particular sport-specific movements place greater stress on one side of the body, thus increasing the disproportions between them [2]. Excessive asymmetry in muscle mass and strength between each side of the body increases the risk of injury and reduces the motor potential of the athlete [3,4,5]. Although many studies have been conducted to quantify and reduce inter-limb asymmetries due to training intervention [2,5,6], the offset training method still has not been explored. The offset training method relies on performing resistance exercises with an asymmetrical position of the external load. Contrary to unilateral exercises with contralateral or ipsilateral external load placement, the offset training method assumes bilateral but asymmetrical position of the external load. The higher external load placed on one side of the body, the greater the postural control as well as the lateral and rotational stability demands on the athlete.

Strength and conditioning coaches use specific approaches that target reductions in muscle imbalances. Symmetrical exercises like the bench press (BP) or the back squats require high movement coordination, balance and stability between both sides of the body for proper execution of the exercise, thus increasing symmetrical demands in its nature. On the other hand, training programs attempting to reduce inter-limb asymmetry commonly include a combination of targeted bilateral and/or unilateral, balance and core exercises [6,7,8]. A study by Bazyler et al. [7] showed that seven-week back squat training lead to significant reductions in inter-limb asymmetry among the weaker but not the stronger group. Furthermore, Núñez et al. [8] indicated that six weeks of unilateral eccentric-overload training improved change of direction turns of 90° in both the dominant and non-dominant leg, while bilateral only in the dominant leg. However, bilateral eccentric-overload training appears to have a higher effect on power output during half-squat tests and jumping performance than the unilateral one. However, both training programs improve muscle volume, yet different lower limb muscle growth occurs. The unilateral eccentric-overload training increased the adductor major and the vastus medialis muscle volume substantially more than the bilateral exercise did, while bilateral training increased the vastus lateralis, vastus intermedius, and the lateral gastrocnemius substantially more than the unilateral loading did. According to the principle of training specificity, variation of symmetrical resistance exercise like the offset method can provide an effective approach to eliminate asymmetry issues without compromising the improvements associated with bilateral exercises.

An electromyography signal (EMG) measures electrical activity in muscle during its contraction and provides easy assessment towards underlying neuromuscular processes that cause muscles to generate force, produce movement and execute a functional task [9,10,11]. Prior studies demonstrated that an asymmetrical position of the external load affects EMG muscle activity during the performance of particular resistance exercises [12,13,14]. Furthermore, in the case of symmetrical exercises such as the BP and back squat, the majority of studies assessing muscle activity are based on EMG data only from one side of the body, often the dominant one [15,16,17]. However, Golas et al. [18] indicated that muscle activity between the right and left side of the upper body during the BP exercise differ significantly, which can lead to inconsistent and incorrect interpretations of obtained results from only one side of the body. Moreover, EMG data can be used to determine asymmetry in muscle activity during the performance of particular exercises. Despite the fact that the offset training method engages core muscles to resist rotation and lateral forces throughout each movement, it also requires the athlete to synchronize both sides of the body and high movement coordination.

Athletes and coaches use multiple types of BP movements to more effectively stimulate the upper-body muscles. A number of studies have examined various issues related to the BP, such as the kinematics and muscle activity of different BP modes [19], unstable exercise conditions [20], fatigue [21], and effectiveness in complex training [22]. However, studies considering muscle activity during asymmetrical position of the external load are limited to unilateral upper [23] and lower body movements [12,14].

To the best of the authors’ knowledge, none of the available studies are related to the changes in muscle activity of the prime movers during the asymmetrically loaded BP with a constant external load. Additionally, to date, there is only one study that compared muscle activity between the right and left side of the upper body during the BP exercise [18]. Thus, the main aim of this study was to compare the changes in muscle activity on both sides of the upper body during different asymmetrical positions of the same external load during the flat BP exercise.

## 2. Materials and Methods

### 2.1. Participants

Ten resistance-trained male students with a minimum of five-years experience in strength training (25.3 ± 2.3 years; 82.9 ± 6.9 kg; 177.8 ± 4.5 cm; 1RM BP: 104.5 ± 8.6 kg; experience: 5.6 ± 1.5 years) participated in this study. All the participants were right-handed. The participants were informed verbally and in writing about the procedures, possible risks and benefits of the tests, and provided written consent before the commencement of the experiment. The measurements were performed in the Strength and Power Laboratory of the Academy of Physical Education in Katowice. The study received the approval of the Bioethics Committee at the Academy of Physical Education in Katowice, Poland (no. 10/2018).

### 2.2. Study Design and Procedure

During the two weeks prior to the experimental session, participants attended 4 familiarization sessions (twice a week) with an asymmetric BP load to eliminate the learning effect. Afterwards, the participants were required to conduct two testing sessions (in the morning between 9:00 and 11:00 a.m.), which were separated by a one-week interval. The first session was used to determine the 1RM load of the flat BP. The second consisted of performing the BP exercise in a random order between an asymmetrically loaded BP (with ~2.5%, ~5% and ~7.5% load differences between sides at 70%1RM), to record the peak muscle activity of the: anterior deltoid (AD), pectoralis major (PM), triceps brachii long head (TB). The participants did not perform any additional resistance exercises for 72 h prior to testing to avoid fatigue. Moreover, subjects were asked to refrain from alcohol, medication and dietary supplements, as well as other ergogenic aids for 24 h prior to testing sessions.

### 2.3. One-Repetition Maximum Strength Test

The maximum strength test to determine 1RM during the flat symmetrically loaded BP was performed one week before the main examinations. The participants cycled on an ergometer for 10 min at an intensity that resulted in a heart rate of 120–140 bpm, followed by a general upper-body warm-up of 10 trunk rotations and side-bends on each side, 10 internal and external rotary movements of the shoulders, 10 push-ups and 5 pull-ups. Afterwards, the participants began a specific warm-up of 15 repetitions at 20% of their estimated 1RM followed by 10 repetitions at 40%1RM, 5 repetitions at 60%1RM and 3 repetitions at 80%1RM of the BP exercise with a 2/0/X/0 tempo of movement and 2-min rest intervals between attempts. Next, the participants performed single repetitions of the BP exercise at their estimated 90%1RM with a 5-min rest interval between successful trials. The load for each subsequent attempt was increased by 2.5 to 10 kg, and the process was repeated until failure. Hand placement on the bar was individually selected (~150% individual bi-acromial distance) and recorded to ensure consistent hand placement during all testing sessions [24]. No weightlifting belts, BP suits or other supportive garments were permitted. All repetitions were performed without bouncing the bar off the chest and intentionally pausing at the transition between the eccentric and concentric phases, as well without raising the hips off the bench. Two experienced spotters were present during all attempts to ensure safety and technical proficiency.

### 2.4. Experimental Session

The general warm-up for the experimental session was identical to the one used during the 1RM test. After warming-up and at the end of the experimental session, the maximal voluntary isometric contraction (MVIC) test of each examined muscle was then recorded to normalize the EMG values according to the SENIAM procedure [25].

Next, the participants performed specific warm-up which was identical to the one used during the 1RM test. Afterward, the subjects performed 7 BP attempts of the BP with symmetric and asymmetric loading at 70%1RM in a random order (symmetric; 2.5%; 5% and 7.5% differences in load between sides, with the accuracy of 0.25 kg, i.e., for 70 kg and 2.5% the asymmetric load equaled 23.25 kg on the right and 26.75 kg on the left side of the bar). Three-minute rest intervals between attempts were adopted. During the tests, the participant received verbal motivation and was spotted by two experienced coaches. The analysis was based on peak muscle activity during each attempt of the BP exercise.

### 2.5. Electromyography

An eight-channel Noraxon TeleMyo 2400 system (Noraxon USA Inc., Scottsdale, AZ, USA; 1500 Hz) was used for recording and analysis of biopotentials from the muscles during each repetition of the flat BP. The activity was recorded for three muscles: PM, AD, TB. Before placing the gel-coated self-adhesive electrodes (Dri-Stick Silver circular sEMG Electrodes AE-131, NeuroDyne Medical, Cambridge, MA, USA), the skin was shaved, abraded and washed with alcohol. The electrodes (11 mm contact diameter and a 2 cm center-to-center distance) were placed along the presumed direction of the underlying muscle fiber according to the recommendations by SENIAM. The EMG signals were sampled at a rate of 1000 Hz. Signals were band pass filtered with a cut off frequency of 8 Hz and 450 Hz, after which the root-mean-square (RMS) was calculated. Following standard procedures, all the electrodes were located on the right and left side of the body. All MVIC test procedures were conducted before and after the experimental session for each side of the body separately, and the highest value was selected for further analysis. Results of MVIC tests were expressed as a percentage of (%MVC). All tests were performed against a fixed multi-press bar. Two maximum isometric contractions were performed for 5-s with a 10-s rest interval between contractions and 2 min between the MVIC evaluation of each muscle according to SENIAM procedures [25]. Positions for the MVIC were selected according to standardized procedures, chosen based on commonly used muscle testing positions for the prime movers of the BP exercise [26]. The TB MVIC test was obtained during the lying triceps extension with 90° elbow flexion, the AD MVIC at 90° seated arm flexion, and the PM MVIC during an isometric BP at 90° elbow flexion.

## 3. Statistical Analysis

Data were presented as the mean and standard deviations. All variables presented a normal distribution according to the Shapiro—Wilk test. Verification of differences between sides of the body (left and right) and load position (symmetric; 2.5%; 5%; 7.5%) was performed using a two-way 2 × 7 (side × load) analysis of variance (ANOVA) with repeated measures for each selected muscle. In the event of a significant main effect, post-hoc comparisons were conducted using the Bonferroni test. Eta-squared (η^2^) was additionally used to determine effect size (ES) in ANOVA tests and the magnitude of differences were considered as small (<0.1), medium (<0.06), or large (>0.14). The statistical significance was set at *p* < 0.05. The 95% confidence intervals were also calculated. All calculations were performed using SPSS (version 25.0; SPSS, Inc., Chicago, IL, USA).

## 4. Results

### 4.1. Anterior Deltoid

**Figure 1:**
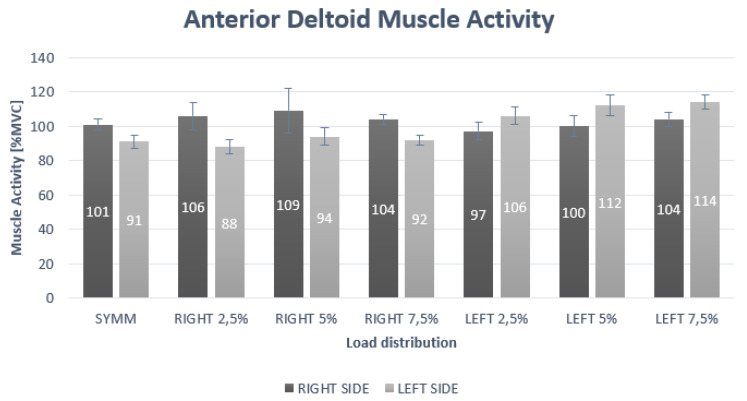
Muscle activity of anterior deltoid under different loading positions.

The two-way repeated measures ANOVA indicated statistically significant interaction for side and load (*p* < 0.01; F = 51.42; η^2^ = 0.72) for the AD muscle (Table 1). The post-hoc analysis for the interaction effect of side × load showed a statistically significant difference at all loads (Table 1). The statistically significant higher muscle activity for the right side was found at symmetric as well as at 2.5%, 5% and 7.5% right asymmetric loading compared to the left side. Furthermore, a statistically significant higher muscle activity for the left side was found at 2.5%, 5% and 7.5% left asymmetric loading compared to right side (Figure 1).

### 4.2. Pectoralis Major

**Figure 2:**
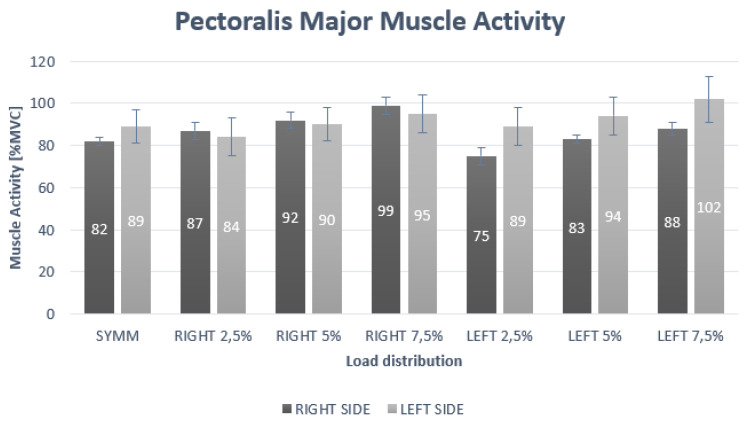
Muscle activity of pectoralis major under different loading positions.

In the case of the PM muscle, the results of the two-way repeated measures ANOVA revealed a significant interaction for side and load (*p* < 0.01; F = 27.11; η^2^ = 0.56) for the PM muscle. The post-hoc analysis for the interaction effect of side × load showed statistically significant differences at symmetric, as well as at 2.5%, 5% and 7.5% left asymmetric loading (Table 1). Significantly higher muscle activity for the left side was found during symmetric loading. Furthermore, a significantly higher muscle activity for the left side was found at 2.5%, 5% and 7.5% in left asymmetric loading compared to the right side (Figure 2).

### 4.3. Triceps Brachii

**Figure 3:**
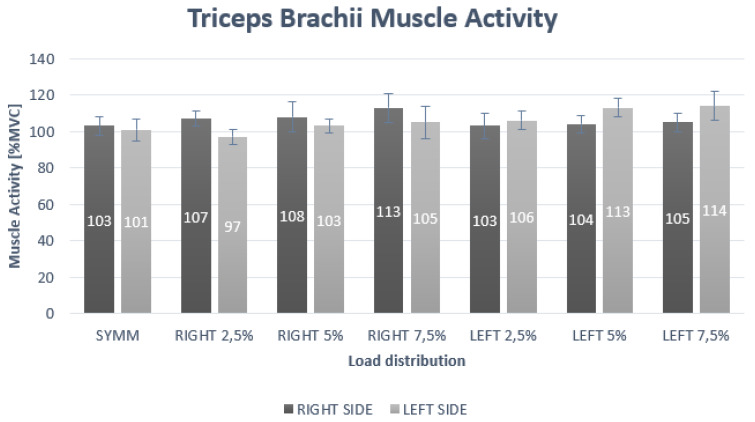
Muscle activity of triceps brachii under different loading positions.

In the case of the TB muscle, the results of the two-way repeated measures ANOVA showed a significant interaction for side and load (*p* < 0.01; F = 10.59; η^2^ = 0.29) for the TB muscle. The post-hoc analysis for the interaction effect of side × load showed statistically significant differences at 2.5% right asymmetric loading and for the 5% left asymmetric loading (Table 1, Figure 3).

## 5. Discussion

The main finding of the study was that asymmetric load BP leads to a statistically significant difference in the pattern of muscle activity between particular sides of the body during the BP exercise, and they are dependent on asymmetrical loading. Muscle activity of AD was significantly higher on the asymmetrically loaded side when compared to the unloaded asymmetrically conditions. In the case of the PM, significantly higher muscle activity of the left PM during all left asymmetric loading was registered, however, there were no differences between the sides when right asymmetric loading was used. Furthermore, muscle activity of the right TB was significantly higher at 2.5% right asymmetric loading and similarly, higher left TB muscle activity was recorded at 5% left asymmetric loading during the BP. Furthermore, the AD and PM muscle activity differed significantly during symmetric BP loading.

To date, most studies focused on instability in resistance training modes, such as free weights instead of a machines [27], dumbbells instead of barbells [19,28], unilateral instead of bilateral exercises [29,30], and the results indicate that those methods effectively increase muscle activity of the prime movers during the performance of particular resistance exercises, even with lower external loading when compared to traditional approaches. A study by Saeterbakken and Fimland [28] showed that the standing dumbbell shoulder press (combining the two instability approaches) demonstrated higher muscle activity of the AD than the standing and seating barbell as well as the dumbbell shoulder presses. Furthermore, Lee et al. [29] noted significantly higher muscle activities during unilateral movement (lunge) in comparison to bilateral movement (squat). To the best of the authors’ knowledge, only Stastny et al. [12] examined the effect of external load position on muscle activity. They showed that the dumbbell-carrying position affects the muscle activity during walking lunges and split squats, with significant differences in the gluteus medius and the vastus lateralis activity, which were associated with the dumbbell-carrying position. However, the study by Stastny et al. [12] investigated unilateral movements, and external loads were not carried on both sides of the body. To date, no previous study considered how asymmetric load position impairs muscle activity during bilateral resistance exercise. The results of the present study indicate that external load placement significantly affects muscle activity patterns during bilateral upper-body movements. Even slight (~2.5%) asymmetric load placement on the non-dominant side of the body leads to significantly greater non-dominant AD muscle activity, while higher asymmetric loading increased (~5%) the TB and PM (~7.5%) muscle activity, without a decrease in the dominant side of the body in comparison to symmetric BP loading. The obtained results may suggest that the use of an offset training method during bilateral resistance exercise may be an attractive and useful approach for the reduction in muscle imbalances and improve the performance of both sides of the body, at the same time.

In the case of the muscle activity patterns during bilateral resistance exercises such as the BP, the majority of studies have reported their results and conclusions only from the dominant side of the body [15,16,17]. However, a study by Golas et al. [18] indicated the necessity of measuring muscle activity on both the dominant and non-dominant sides of the body, especially when an investigation is conducted on competitive-strength athletes, and very heavy loads are used. The results of the present study confirm that statement among resistance-trained male students. Muscle activity measurements performed in the present study showed significant differences between the left and right AD, as well as between the left and right PM during symmetric BP loading. It should be mentioned that AD muscle activity was significantly higher in the dominant when compared to the non-dominant side of the body. On the contrary, PM muscle activity was significantly lower in the dominant than the non-dominant side of the body. These results show that the body laterality significantly affects the movement pattern with significant differences in selective activation of muscles during the BP exercise. The higher AD muscle activity with lower involvement of the PM on the dominant side of the body may indicate that the PM is a supportive prime-mover on the dominant side of the body during the flat BP exercise. Explanations of such results may be related to the role of the dominant limb, which optimizes the dynamic features of movement, whereas the non-dominant limb is specialized in stabilizing and correcting the movement tasks [31]. Considering the research of Golas et al. [18], and the results of this study, it seems advisable to analyze the muscle activity from both the non-dominant and dominant sides of the body.

Certain study limitations should be acknowledged. The external structure of the movement (i.e., kinematics and movement torque) was not investigated. Additionally, the activity of antagonist and core muscles was not evaluated. Furthermore, even though the participants in this study were resistance-trained men, the sample size was relatively small. Moreover, only one external load (70%1RM) was used during the evaluations.

## 6. Conclusions

The present study compared the muscle activity of selected muscles during symmetric and chosen asymmetrically-loaded BP at 70%1RM among resistance-trained males. The results indicate that the offset training method significantly affects muscle activity during the BP. Additionally, compared to the symmetrical BP, the asymmetrical load position on the non-dominant body-side leads to significantly greater prime mover activity on that side of the body, without a decrease on the dominant side. In future studies, additional attention should be paid to other external loads, which may significantly affect muscle activity and test results.

## 7. Practical Implications

The obtained results provide practical implications for strength and conditioning coaches and athletes, which may suggest that the use of the offset training method during bilateral resistance exercises may be an effective and simple approach for improving the performance of both body-sides with simultaneous reductions in muscle imbalances. Based on higher muscle activity during asymmetric BP loading in this study, it can be expected that the offset training method provides additional exercise effects in BP prime movers for physical training and during re-education after injury.

## Figures and Tables

**Table 1:** Post-hoc comparisons between both sides of the body for all measured loads.

Side		Symm	Right_2.5%_ (95% CI)	Right_5%_ (95% CI)	Right_7.5%_ (95% CI)	Left_2.5%_ (95% CI)	Left_5%_ (95% CI)	Left_7.5%_ (95% CI)	*p*	F
Anterior Deltoid [%] MVC										
Right		101 ± 3 (98–103)	106 ± 8 (100–112)	109 ± 13 (100–118)	104 ± 3 (102–106)	97 ± 5 (93–101)	100 ± 6 (96–104)	104 ± 4 (102–107)	<0.001	51.42
Left		91 ± 4 (88–94)	88 ± 4 (85–91)	94 ± 5 (90–98)	92 ± 3 (89–94)	106 ± 5 (102–110)	112 ± 6 (108–117)	114 ± 4 (111–117)		
Right vs. left	*p*	<0.001 *	<0.001 *	<0.005 *	<0.001 *	<0.001 *	<0.001 *	<0.001 *		
	ES	0.67	0.67	0.37	0.8	0.45	0.5	0.61		
Pectoralis major [%] MVC										
Right		82 ± 2 (81–84)	87 ± 4 (84–90)	92 ± 4 (89–95)	99 ± 4 (96–101)	75 ± 4 (72–78)	83 ± 2 (82–85)	88 ± 3 (86–90)	<0.002	18.81
Left		89 ± 8 (83–96)	84 ± 9 (77–90)	90 ± 8 (84–96)	95 ± 9 (88–101)	89 ± 9 (82–95)	94 ± 9 (88–101)	102 ± 11 (94–110)		
Right vs. left	*p*	0.032 *	0.304	0.572	0.325	0.001 *	0.004 *	0.002 *		
	ES	0.26	0.04	0.03	0.08	0.5	0.42	0.43		
Triceps brachii [%] MVC										
Right		103 ± 5 (100–107)	107 ± 4 (105–110)	108 ± 8 (102–113)	113 ± 8 (107–119)	103 ± 7 (100–107)	104 ± 5 (100–107)	105 ± 5 (102–109)	<0.001	10.58
Left		101 ± 6 (97–105)	97 ± 4 (94–100)	103 ± 4 (99–106)	105 ± 9 (98–111)	106 ± 5 (103–110)	113 ± 5 (109–117)	114 ± 8 (108–119)		
Right vs. left	*p*	0.492	<0.001 *	0.184	0.134	0.054	<0.001 *	0.059		
	ES	0.03	0.61	0.14	0.18	0.06	0.45	0.31		

Data are presented as mean ± standard deviation and 95% confidence interval (95% CI); ES = effect size; %MVC = % of maximum voluntary contraction; Symm = symmetrical load; Right_XX%_ = XX% asymmetrical load on the right side of the bar; Left_XX%_ = XX% asymmetrical load on the left side of the bar; * significant differences *p* < 0.05.
